# Effect of monovalent cations on the kinetics of hypoxic conformational change of mitochondrial complex I

**DOI:** 10.1016/j.bbabio.2015.05.012

**Published:** 2015-10

**Authors:** Anna Stepanova, Alba Valls, Alexander Galkin

**Affiliations:** a Queen's University Belfast, School of Biological Sciences, Medical Biology Centre, 97 Lisburn Road, Belfast BT9 7BL, UK; b N.K. Koltzov Institute of Developmental Biology, Russian Academy of Sciences, 26 Vavilova Str., Moscow 119334, Russia; c University of Barcelona, Faculty of Biology, Diagonal, 643, 08028 Barcelona, Spain

**Keywords:** A/D, active/de-active transition, BSA, bovine serum albumin, EIPA, 5-(N-Ethyl-N-isopropyl)amiloride, FCCP, carbonyl cyanide 4-(trifluoromethoxy)phenylhydrazone, HAR, hexaammineruthenium(III)-chloride, Q_1_, 2,3-dimethoxy-5-methyl-6-(3-methyl-2-butenyl)-1,4-benzoquinone, ROS, reactive oxygen species, SMP, bovine heart submitochondrial particles, TMH, transmembrane helix, Mitochondrial complex I, NADH:ubiquinone oxidoreductase, A/D transition, Conformational change, Sodium, Ischemia/reperfusion

## Abstract

Mitochondrial complex I is a large, membrane-bound enzyme central to energy metabolism, and its dysfunction is implicated in cardiovascular and neurodegenerative diseases. An interesting feature of mammalian complex I is the so-called A/D transition, when the idle enzyme spontaneously converts from the active (A) to the de-active, dormant (D) form. The A/D transition plays an important role in tissue response to ischemia and rate of the conversion can be a crucial factor determining outcome of ischemia/reperfusion. Here, we describe the effects of alkali cations on the rate of the D-to-A transition to define whether A/D conversion may be regulated by sodium.

At neutral pH (7–7.5) sodium resulted in a clear increase of rates of activation (D-to-A conversion) while other cations had minor effects. The stimulating effect of sodium in this pH range was not caused by an increase in ionic strength. EIPA, an inhibitor of Na^+^/H^+^ antiporters, decreased the rate of D-to-A conversion and sodium partially eliminated this effect of EIPA. At higher pH (> 8.0), acceleration of the D-to-A conversion by sodium was abolished, and all tested cations decreased the rate of activation, probably due to the effect of ionic strength.

The implications of this finding for the mechanism of complex I energy transduction and possible physiological importance of sodium stimulation of the D-to-A conversion at pathophysiological conditions *in vivo* are discussed.

## Highlights

• The active/dormant (A/D) transition of complex I is affected by monovalent cations.
• Na^+^ increases the rate of the D/A conversion at neutral pH.
• Lithium and caesium decrease D/A transition at all tested pH
• Matrix ion balance may influence the rate of the activation of the enzyme in situ.

## 1 Introduction

Proton-translocating NADH:ubiquinone oxidoreductase (EC 1.6.5.3, complex I or Type I NADH dehydrogenase) is the most complex enzyme of the mitochondrial respiratory chain. Complex I is responsible for the oxidation of matrix NADH by membrane ubiquinone (Q) and transport of four protons across the inner membrane, contributing to the proton-motive force used for ATP generation [1–4]. The enzyme is also thought to be a major contributor to the production of reactive oxygen species in the respiratory chain [5–7]. The atomic structure of the entire complex I from *Thermus thermophilus* (16 subunits) suggests a unique mechanism, with redox energy of electron transfer driving proton translocation via long-range conformational changes [3,8]. Mammalian complex I consists of at least 45 subunits [9], of which more than 30 so-called “accessory” subunits are not directly involved in catalysis and whose functions are still unclear. Recently published overall architecture of mitochondrial complex I [10,11] suggests similar organisation of the catalytic core and principle of operation via long-range conformational changes to the prokaryotic enzyme. There are no redox cofactors in the membrane domain of the enzyme, and transmembrane subunits involved in the proton translocation are far from the interface with the hydrophilic domain. The junction between these two domains is a key area where the redox energy of electron transfer from terminal cluster N2 to ubiquinone molecule is transduced and transmitted into the membrane part and used for translocation of protons. Seven mitochondrial-encoded membrane core subunits form four sets of putative channels directly involved in proton translocation [10].

In bovine enzyme, three mitochondrially encoded subunits, ND5, ND4, and ND2 (NuoL, M, and N in prokaryotes), are homologous to each other [12] and to an Mrp-type Na^+^/H^+^ antiporter from *Bacillus halodurans* [13] and are often referred to as antiporter-like subunits. This homology gave rise to the discussion as to whether or not, in addition to protons, there could also be other ions, namely, sodium, translocated by mitochondrial complex I in a redox-dependent manner [14–17].

The catalytic properties of mitochondrial complex I are not simple (for a review, see [18,19]). Two catalytically and structurally distinct forms exist in any given preparation of the enzyme: one is the fully competent, so-called active A-form, and the other is the catalytically silent, dormant, D-form. Reversible A/D transitions have been described in mammalian [20] and other eukaryotic complex I [21] and have been reviewed in detail [19]. After incubation of idle enzyme preparations at physiological temperatures when catalytic turnover cannot occur, complex I undergoes deactivation. In the presence of both NADH and ubiquinone, the D-form is converted back to the A-form as a result of the slow turnover(s) of the enzyme (first-order rate constant, k_D → A_ ≈ 1 min^− 1^). *In situ*, in conditions of limited oxygen concentration (*i.e.*, ischemia) all mitochondrial redox components including ubiquinone undergo almost complete reduction due to the slowing of cytochrome *c* oxidase. In such situations, the steady-state equilibrium of the A↔D transition is shifted to the right. In highly metabolising tissues such as heart and brain, enzyme readily deactivates in minutes during ischemia and is reactivated upon reperfusion [22–24].

The exact molecular mechanism causing the hypoxic A/D transition is unknown. It has been shown that A to D transition involves a conformational change of a mitochondrially encoded subunit ND3. This conformational change exposes a critical Cys-39 in the first hydrophilic loop of ND3 and is accompanied by a concerted change in the exposure of ND1 subunit and nuclear-encoded 39 kDa subunit (NDUFA9) [25–27]. ND1, ND3, and NDUFA9 subunits are located at the junction between the hydrophilic and membrane arms of complex I. ND1 and ND3 are directly involved in the formation of the quinone binding pocket [3,10].

It is accepted that electron transfer in mitochondrial and bacterial complex I is coupled with primary translocation of protons [1,2,4,15]. Steuber's group initially reported that complex I from enterobacterium *Klebsiella pneumoniae* pumps sodium ions rather than protons [28]. This claim was later challenged by [29], clearly showing that NADH-dependent sodium translocation in these experiments was probably due to contamination of complex I preparations by the sodium-motive NQR-type enzyme. Despite the publication of redox-dependent sodium translocation by mitochondrial enzyme [16], no indications suggesting that complex I functioned as a sodium pump were found [15,30]. In addition, a potential Na^+^/H^+^ antiporter activity was demonstrated for NADH-oxidase activity of subbacterial particles from *Escherichia coli* and *Rhodothermus marinus* [31,32]. Later, Na^+^/H^+^ exchange catalyzed by the D-form of the bovine enzyme was shown on proteoliposome-reconstituted enzyme and submitochondrial particles (SMP) [33].

Structural [34–36] and functional studies [17,31,33,37] suggest a strong possibility of a specific interaction between complex I and sodium ions affecting the A/D behavior of the enzyme. Putative sodium-binding sites are located at a long distance from the area of the enzyme where A/D-dependent change takes place. Taking into account long-range conformational change during catalytic turnover, the impact of cation binding at the membrane part of the enzyme on the mobility/rigidity of subunits involved in A/D transition cannot be excluded. In addition, slow turnover-dependent A to D conversion might involve dissociation of bound sodium ions from antiporter-like subunits in order for the enzyme to initialize H^+^-translocation machinery. *In situ*, at physiological conditions of the mitochondrial matrix, complex I operates at high osmolarity and ionic strength in the presence of different cations, such as K^+^, Na^+^, which may modulate the dynamics of the A/D transition.

Thus, it seems plausible to investigate the effect of alkali cations on the kinetics of the A/D transition of bovine complex I. In the present study, we demonstrated that alkali cations had a minor effect on the absolute rate of NADH-dependent reactions of the A-form, but significantly affected the rate of the D-to-A transition. Unlike other monovalent cations, sodium showed a stimulating effect on the D-to-A transition, which was pronounced only at neutral pH. At higher pH, all tested cations including sodium decelerated the rate of activation.

Whether or not Na^+^ ions can be translocated by mitochondrial complex I was out of the scope of the presented paper since we focused on the effect of alkali cations on the kinetics of A/D transition of the enzyme. Our results provide new information about the mechanism of A/D transition of mammalian complex I and are aimed to identify the link between sodium ions and conformational change. This will help to define possible modes of fine regulation of the enzyme's activity during ischemia-reperfusion-associated conditions.

## 2 Materials and methods

Bovine heart SMP were prepared according to standard procedure [9] and stored in liquid nitrogen.

To prepare SMP in which complex I is in the D-form, SMP were resuspended to 5 mg/ml in SET buffer pH 8.5 composed of 0.25 M sucrose, 0.2 mM EDTA, 50 mM Tris-HCl and incubated at 35 °C for 30–60 minutes under constant shaking. This treatment resulted in almost complete deactivation of complex I (> 90%). To obtain SMP containing the A-form of the enzyme, suspension of SMP (5 mg/ml) was treated with a NADH regenerating system as described previously [38]. Alternatively, the enzyme was activated by a 10 μM pulse of NADH added directly to the spectrophotometric cuvette, and then full NADH oxidase or NADH:Q_1_ oxidoreductase was assayed [38].

The enzymatic activities were assayed at 25 °C spectrophotometrically (Varian Cary 4000) as a decrease in absorption at 340 nm (ε_340nm_ = 6.22 mM^− 1^ cm^− 1^) with 165 μM NADH in SET medium containing 10–50 μg of protein/ml SMP. For the measurements of NADH:Q_1_ or NADH:HAR oxidoreductase activity, SMP were assayed in the presence of 1 mM cyanide with the addition of 40 μM Q_1_ or 1 mM HAR, respectively. Chloride salts of alkali metals were used in all experiments. Initially, all alkali metals were tested, but for the sake of clarity, only results for three cations, *i.e.*, lithium, sodium, and cesium, are presented.

All chemicals were purchased from Sigma. Protein content was determined with a BCA assay. Reported values are the mean ± SD, which were analyzed by the two-sample t-test to determine statistically significant differences of means among groups (three or more independent experiments). The experimental details are described in the legends to figures.

## 3 Results

### 3.1 The effect of monovalent cations on the NADH-dependent activities of complex I.

**Fig. 1:**
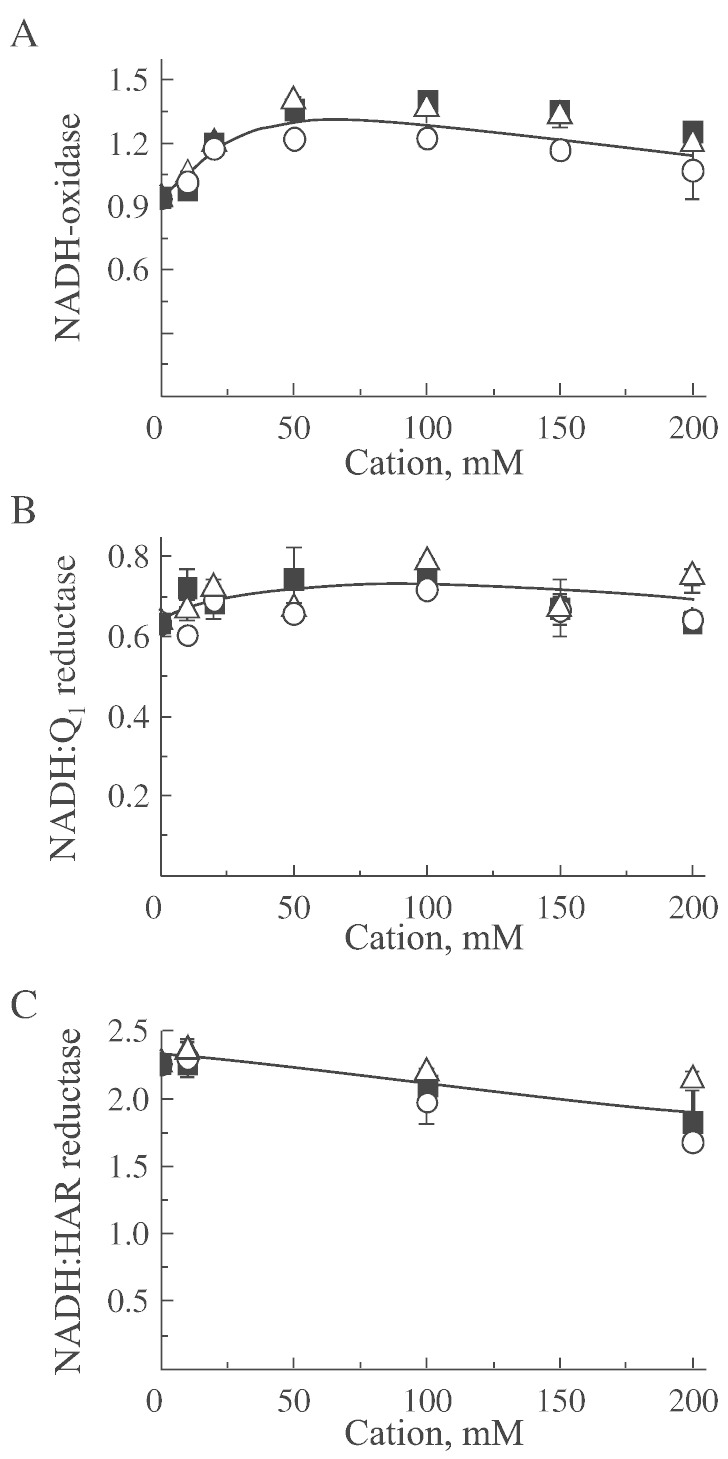
The effect of different monovalent cations on NADH-dependent reactions catalyzed by fully active complex I in bovine heart SMP in SET buffer at pH 7.5. Activities were measured as described under “Materials and Methods” in the presence of different concentrations of lithium (○), sodium (Δ), and cesium (■). A, NADH-oxidase, B, NADH:Q_1_ oxidoreductase, C, NADH:HAR oxidoreductase. Values are given in μmol NADH × min^− 1^ × mg^− 1^.

Fig. 1 presents the effects of lithium, sodium, or cesium on various NADH-dependent catalytic activities of the A-form of complex I from bovine heart SMP. Results with salts of other alkali metals such as potassium and rubidium were similar (not shown). NADH-oxidase activity was increased by 30–40% in the presence of 100 mM ions most likely due to the effect of ionic strength on interactions of complex IV with cytochrome *c* in SMP (Fig. 1A). As expected, monovalent cations exerted little effect on NADH:Q_1_ reductase activity of complex I (Fig. 1B).

HAR is an artificial electron acceptor that most likely accepts electrons directly from the redox center(s) downstream of the nucleotide binding site of complex I. This activity is not sensitive to hydrophobic specific inhibitors of complex I such as rotenone and it is not dependent on the A/D state of the enzyme. There was a small decline in NADH:HAR reductase reaction with increasing ionic strength (Fig. 1C).

### 3.2 Kinetics of activation of the D-form of complex I

**Fig. 2:**
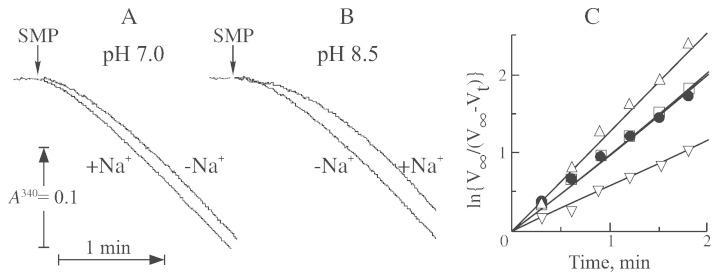
Effect of sodium on kinetics of the NADH-oxidase reaction catalyzed by the D-form of complex I from bovine SMP. Representative traces of NADH oxidation by SMP containing only the D-form of complex I. The reaction was initiated by addition of SMP (17.5 μg/ml) to 1 ml of SET buffer pH 7.0 (A) and 8.5 (B) containing 150 μM NADH in the absence (1, 2) and in the presence (3, 4) of 200 mM sodium. (C) Determination of the first-order rate constant of activation (k_D → A_) in the NADH-oxidase reaction catalyzed by the D-form of the enzyme. Linear transformation traces from the A and B: trace 1 pH 7, no sodium (●), trace 2 pH 7.0, 200 mM sodium (Δ), trace 3, pH 8.5, no sodium (□), trace 4, pH 8.5, 200 mM sodium (∇).

As previously shown, the A- and D-forms of the enzyme in SMP demonstrate different kinetic behavior in physiological NADH-oxidase or NADH:Q_1_ reductase reactions [20,39]. In these conditions, when the reaction was started by SMP containing complex I in the D-form, both NADH-oxidase and NADH:ubiquinone reductase were catalyzed with a noticeable lag phase. Duration of the lag phase (*i.e.*, the rate of activation) was found to be dependent on the presence of monovalent cations. In Fig. 2, the time course of the NADH-oxidase reaction is shown in the presence and absence of 200 mM sodium at pH 7.0 and 8.5 (Fig 2, A and B).

Turnover-dependent activation of complex I during NADH oxidation follows first-order kinetics [20], and therefore rate constant (k_D → A_) can be derived from the semilogarithmic plots as shown in Fig. 2C. It was clear that at pH 7.0, addition of sodium significantly increased the rate constant of activation (from 0.93 ± 0.08 to 1.23 ± 0.01 min^- 1^). The opposite effect was observed at pH 8.5, when in the presence of sodium k_D → A_ was decreased (from 0.82 ± 0.06 to 0.64 ± 0.07).

**Fig. 3:**
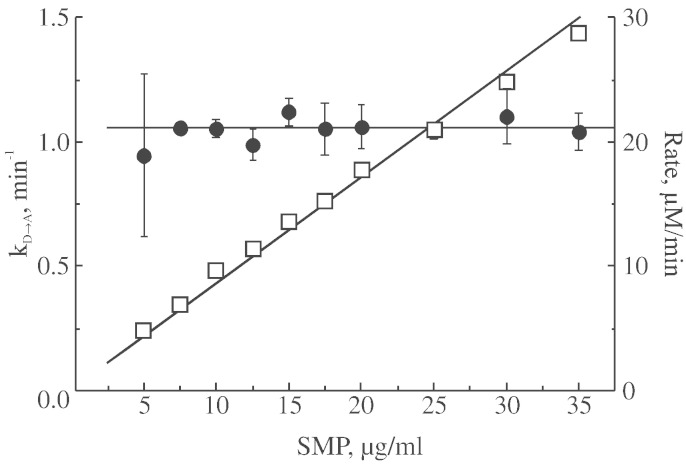
Dependence of activation rate constant (k_D → A_) and rate of the NADH-oxidase reaction on the amount of the enzyme present in the assay (pH 7.5). Activation rate constant (●) was determined as in Fig. 2B. Reaction was initiated by addition of SMP containing complex I in the D-form to a standard assay supplemented with 150 μM NADH and final rate of the NADH-oxidase reaction was assessed after full activation of the enzyme (□).

In order to eliminate any pitfalls and artefacts of calculation of activation rate constant (due to the possible alteration of the absolute rate at higher ionic strength or different pH), we determined the value of k_D → A_ in a wide interval of enzyme concentration. As shown in Fig. 3, almost seven-fold increase in concentration of the enzyme in an assay resulted in proportional increase of the NADH-oxidase reaction rate. However, this did not have any effect on the constant calculated from the activity traces using semilogarithmic plots as in Fig. 2B. Therefore, possible variations in the absolute activity, caused by addition of salts or inhibitors, would not affect the accuracy of our calculation approach.

**Fig. 4:**
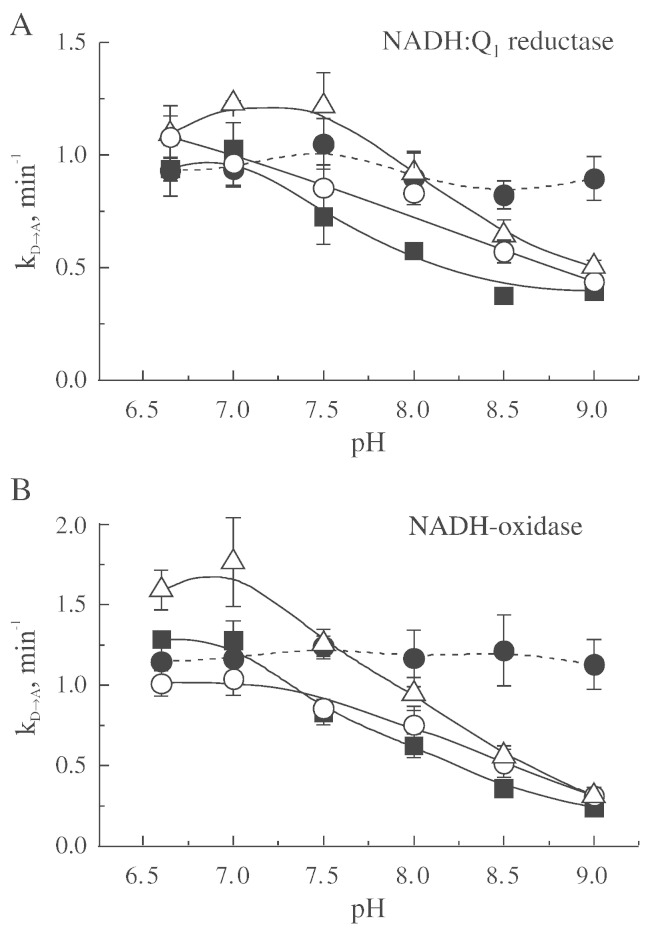
The pH-dependence of the rate constant of activation (k_D → A_) of the enzyme in NADH:Q_1_ reductase (A) and NADH-oxidase (B) reaction in the absence (●) or presence of 200 mM lithium (○), sodium (Δ) and caesium (■).

The duration of the lag phase in the absence of cations was found to be pH-independent, indicating no effect of pH on the value of the k_D → A_ in both NADH:Q_1_ reductase and NADH-oxidase reactions when measured in SET buffer (Fig. 4). However, this changed drastically upon addition of monovalent cations. At neutral pH, only sodium stimulated the rate of the D-to-A transition by 50% and 30%, in NADH:Q_1_ reductase and NADH-oxidase reactions, respectively. At the same time, the opposite effect was observed in more alkaline medium (Fig. 4). In our initial studies, we found that only near-neutral pH (< 7.5) the nature of the cation had a substantial effect on the rate constant of activation both in NADH-oxidase and in NADH:Q_1_ reductase (Fig. 4).

**Fig. 5:**
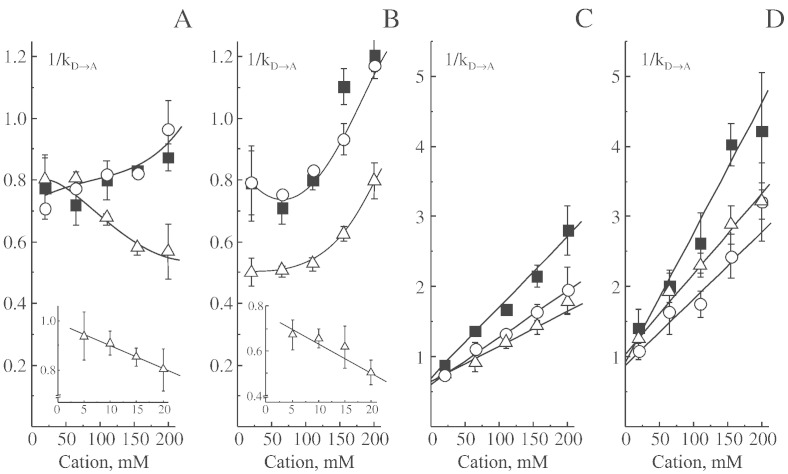
Dependence of the apparent rate constant of activation (k_D → A_) in the NADH:Q_1_ reductase reaction on concentration of lithium (○), sodium (Δ), and cesium(■) at pH 7, 7.5, 8.5, and 9.0 (A, B, C, and D, respectively). Dependence of k_D → A_ at low concentration of sodium at pH 7.0 and 7.5 are shown at the insets to A and B.

The dependence of apparent rate constant of activation (k_D → A_) on concentration of monovalent cations at different pH is shown in Fig. 5. At neutral pH minor effects of lithium and cesium on the k_D → A_ were observed, while addition of sodium resulted in a clear increase in rates of activation. At higher pH the stimulating effect of sodium was abolished.

Values of apparent affinity of the D-form for alkali ions were determined from linear reciprocal plot at several pH (Fig. 5). The linear plots of l/k_D → A_ versus ion concentration were obtained and apparent dissociation constant (K_D_) was calculated from the intercepts of the straight lines with the abscissa [40]. The experimental data shown in Table 1 confirms that the apparent affinity of the deactivated enzyme for alkali ions increases as the pH of the assay medium increases. Due to the apparent stimulation effect of sodium ions at pH < 7.5 (Fig. 5, A and B), only data for pH interval 8.0–9.0 are shown for this ion (Table 1).

Rate constant for complex I activation (k_D → A_) in the presence of 200 mM monovalent cations also depends strongly on pH of the medium in the interval of 7.0–9.0 (Fig. 5 and Table 1).

In our experiments, the duration of the lag phase seen in the absence of any cations was found to be pH-independent (Fig. 4). However, our finding contradicts earlier data [40,41], in which activation rate constant was found to be much slower at the alkaline pH. We tested pH dependence of the D-to-A conversion using the exact assay conditions as in the original paper [40] (0.25 M sucrose, 50 mM HEPES, 0.1 mM EDTA, 5 mM malonate, 1 mg/ml BSA for SMP deactivation and 0.25 M sucrose, 0.1 mM EDTA, 0.1 μM FCCP for measurements). The apparent rate of activation in this condition was not found to be affected by alkalization of the measuring medium (4.7 min^− 1^ at the pH interval from 7.0–9.0).

**Fig. 6:**
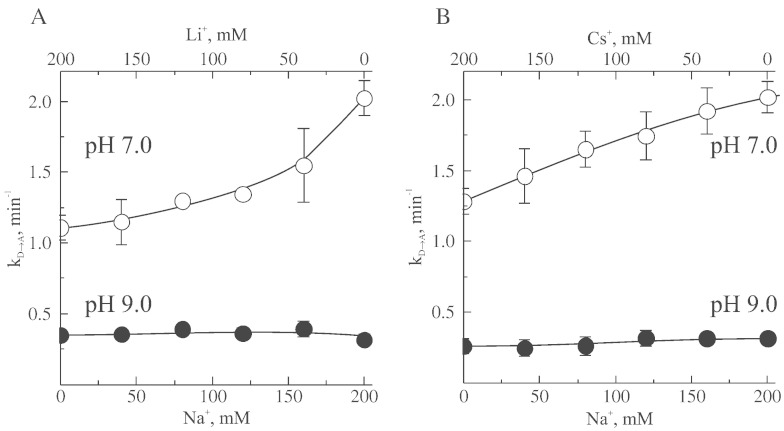
Values of apparent activation rate constant (k_D → A_) in the NADH:Q_1_ reductase reaction in the presence of different concentrations of sodium at pH 7.0 and 9.0 (open and closed circles respectively). Total ionic strength of the medium was kept constant using a combination of sodium with either lithium (A) or caesium (B) ions.

Next, we examined the effect of sodium on D-to-A transition at different pH using a combined system of Na^+^/Li^+^ or Na^+^/Cs^+^, changing concentrations of both ions while keeping total ionic strength constant (200 mM). If the stimulating effect of sodium (Fig. 5A) was independent of ionic strength, then in the system where total concentration of both cations [Na^+^+ M^+^] is kept constant, the increase in sodium content should correlate with increase in k_D → A_ values only at pH < 7. As shown in Fig. 6, an increase in the concentration of sodium in the presence of another alkali ion resulted in stimulation of D-to-A change, *i.e.*, increase in the values of apparent k_D → A_ rate constant. This effect was only observed at pH 7 and disappeared at more alkaline pH > 8.0.

Data presented at Figs. 5 and 6 may be explained by two opposite effects of the cations at different pH. At pH < 7.5, sodium increased the rate of D-to-A conformational change, and this phenomenon disappeared with the increase in pH. (Fig. 5, A and B). At more alkaline pH, all tested cations decreased the rate of activation, due to the effect of ionic strength clearly evident at pH > 8.0.

### 3.3 Effect of EIPA on activation of the D-form of complex I.

**Fig. 7:**
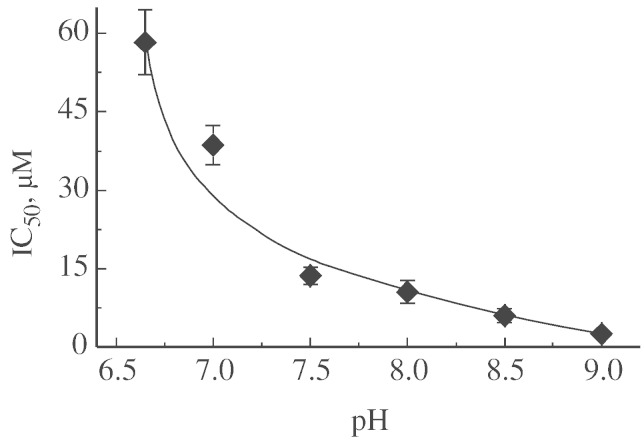
pH dependence of IC50 for EIPA in NADH-oxidase reaction of SMP containing the A-form of complex I. SMP (20 μg/ml) were incubated for 5–7 min in SET buffer with various concentration of the inhibitor directly in the spectrophotometer cuvette and reaction was initiated by addition of 165 μM NADH.

**Fig. 8:**
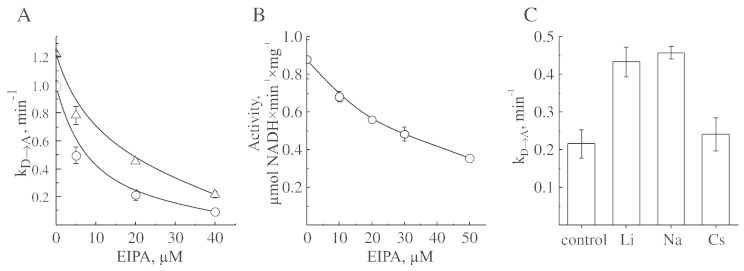
Effect of cations on the inhibition of A/D transition by EIPA. SMP containing the D-form of complex I were incubated for 5 min with inhibitor in a SET medium (pH 7.0) directly in the spectrophotometer cuvette and NADH-oxidase reaction was initiated by addition of 165 μM NADH. A, effect of EIPA on the rate of D-to-A transition in the presence (Δ) and absence of sodium (○), B, effect of EIPA on the activity of the enzyme, C, effect of various alkali cations (200 mM) on the activation rate of enzyme in the presence of 20 μM EIPA.

It has been shown that the function of complex I can be affected by inhibitors of Na^+^/H^+^ antiporters such as EIPA [32,37,42]. The effectiveness of the inhibitor towards Na^+^/H^+^ exchanger increases with decreasing in sodium concentration, providing evidence for a shared binding site for amiloride and sodium [43,44]. Therefore, it was of interest to study the effect of EIPA on the D-to-A transition in the presence and absence of sodium in the conditions where this cation showed a stimulating effect. The inhibitory effect of EIPA on NADH:Q_1_ activity of bovine complex I was found to be strongly pH-dependent with IC_50_ decreased around five-fold at the pH interval from 7.0–8.0 (Fig. 7). Since the stimulating effect of sodium was observed only at neutral pH, we tested the effect of EIPA on complex I at pH 7.0. Addition of EIPA affected the activity of the enzyme and decreased the rate of D-to-A conversion (Fig. 8, A and B). Addition of sodium partially abolished the inhibitory effect of EIPA on the D-to-A transition (Fig. 8, A and B), but had very little effect on the activity (Fig. 8B). Inhibitory potency of EIPA was exactly the same in the presence and the absence of sodium (not shown). Interestingly, both sodium and lithium decreased the inhibitory effect of EIPA on the D-to-A transition, but other cations including cesium did not show a protective effect (Fig. 8C). At pH 7.0 the 20 μM EIPA lowered k_D → A_ from 1.00 ± 0.04 to 0.22 ± 0.04 min^− 1^ in the absence of alkali cations, and addition of sodium or lithium increased the value for EIPA-treated enzyme about two-fold (Fig. 8C).

## 4 Discussion

Alkali metal ions are present in high concentrations in the mitochondria, such that estimated ionic strength in the matrix is equivalent to 150 mM KCl [45,46]. Therefore, it is reasonable to assume that alkali cations may be involved in modulation of enzymatic function. Similarity of subunits ND5, ND4, and ND2 to Na^+^/H^+^, antiporter and experimental data available to date [31,33,34,36,37] indicate that sodium might be binding specifically to mitochondrial complex I, thus affecting its catalytic behavior.

In our initial studies, we observed very minor effects of alkali cations on NADH-dependent activities of mitochondrial complex I in bovine SMP at pH 7.5. Only a minor increase in NADH-oxidase reaction was found, most likely indicating activation of the cytochrome *c* oxidase in inside-out SMP at higher ionic strength [47,48]. Unlike in prokaryotic enzyme [49,50], NADH:ubiquinone reductase activity of bovine complex I in SMP in neutral medium was not affected by ionic strength up to 200 mM salt.

It has been shown that activation of the enzyme (D-to-A transition) involves subunits ND3, ND1, and most likely 39 kDa [27] and, based on the crystal structure, subunit 49 kDa (NDUFS2). Slow concerted movement of their loops results in formation of the quinone binding site in the A-form and could also reflect a critical energy-transducing step during steady-state catalytic turnover. Our observations support this hypothesis. Steady-state NADH:ubiquinone reductase activity of the enzyme has a very broad pH optimum [19], and in our experiments, the duration of the lag phase seen in the absence of any cations was also found to be pH-independent. This observation contradicts earlier finding from Vinogradov's group [40]. A possible explanation for this discrepancy might be the different lipid composition of SMP membrane from each author’s lab due to the young age of cattle used as well as seasonal variations [51,52]. It is known that membrane fatty acids affect the dynamics of A/D transition in a pH-dependent manner [53].

We show that alkali cations have strong and pH-dependent effects on the kinetics of the conformational A/D transition of the bovine complex I. We observed that at pH > 7.5 all tested alkali metal cations decelerate the rate of D-to-A conversion; however, this inhibitory effect is probably unspecific. It has been well recognized that membrane surface charge density is an important factor in many of the functions of biological membrane systems. We suggest that at higher pH the deprotonation of membrane lipids or negatively charged TMH5 loop of ND1 results in more effective interactions with cations, and therefore shielding favorable electrostatic interactions involved in D-to-A conformational change [11].

While none of the cations tested have effects on activation of the D form in alkaline conditions, at neutral pH, only sodium ions show a stimulating effect. This was due to the specific effect of increase in sodium concentration and not due to the increase of total ionic strength of the medium. Control experiments were carried out in which part of the sodium was substituted by either lithium or cesium without changing the total ionic strength of the medium. This substitution decreased the rate of the D-to-A transition, and the stimulating effect of sodium was abolished. Conversely, increasing the sodium content in the combined system significantly accelerates the D-to-A transition.

It is also possible that the observed effect is related to the affinity of ions to the putative binding site at the antiporter-like subunits of the enzyme. Stimulatory effect of sodium in the combined system reflects higher affinity of the binding site(s) to sodium and therefore elevated occupancy of the enzyme by this ion at neutral pH.

Therefore, we conclude that there are two opposite effects of the cations. One is a sodium-specific stimulation of the enzyme D → A transition (increase in k_D → A_ value) occurring at neutral pH; and the other is unspecific deceleration of D-to-A conversion by ionic strength observed for all cations at more alkaline pH (> 7.5). Even if sodium-specific activation takes place at alkaline pH, this would be masked by the inhibitory effect of ionic strength.

Although we found that variation in both pH and ionic strength are important factors in the process of the D-to-A conformational change of mitochondrial complex I, quantitatively assessing their role in modulating electrostatic energies is challenging [54]. Our results suggest that interaction of membrane subunits with lipid molecules is an important factor determining the dynamics of A/D conversion. It is in conjunction with our previous identification of two membrane subunits ND3 and ND1 found to be directly involved into A/D transition [26]. Subunit 39 kDa (NDUFA9) [25,27] is also associated with this conformational change and it was thought to be anchored to the membrane [55,56]. Although recent structural studies did not show any transmembrane helices in this subunit, it is in fact located very close to the membrane surface [10].

Another important observation is the specific effect of sodium on the process of A/D transition of the enzyme. As mentioned before, the structural rearrangements during de-activation of complex I include subunits located at the junction between hydrophilic and hydrophobic domains, in the region of the quinone binding site. Three antiporter-like membrane subunits ND5, ND4, and ND2 are located at significant distance from the site of these changes [3,10]. Therefore, the question of how potential binding of sodium ion(s) would affect the mobility/rigidity of the subunits near the Q-binding site is an open one. During catalytic turnover, redox energy of electron transfer released at the quinone-binding site should be somehow transduced and delivered to membrane part. Taking into account reversibility of the NADH:quinone oxidoreductase reaction, it is reasonable to expect a possibility of long-distance transmission of a change within distal membrane subunits towards the hydrophilic domain. Consequently, binding of sodium at the distal antiporter-like subunit(s) can potentially influence dynamics of the entire coupling machinery including area of quinone-binding site, therefore affecting the process of A/D transition. It was suggested that long transverse helix HL of ND5, a proposed coupling element of proton translocation, coordinates conformational changes by linking discontinuous transmembrane helices between the ND5, ND4, and ND2 [57,58]. Whether or not activation of the enzyme during catalytic turnover is associated with energy-dependent ion translocation is still unclear. At the same time, although we did not observe any influence of monovalent cations on absolute NADH:ubiquinone activity of the enzyme, a direct effect of ions on the process of ubiquinone binding to the enzyme cannot be excluded.

Our data obtained from EIPA-inhibited enzyme confirmed sodium-specificity of this stimulating effect. At neutral pH, EIPA is a relatively weak inhibitor of NADH:Q_1_ oxidoreductase activity of the bovine enzyme (IC_50_ ~ 40 μM) and also decelerates D-to-A transition. Addition of sodium abates the inhibitory effect of EIPA. Taking into account that the guanidinium group of amiloride and a hydrated sodium ion are of similar size and shape [59], binding sites of sodium and EIPA in Na^+^/H^+^ antiporter molecule may overlap [43,44]. Therefore, it is not surprising that in the presence of sodium (or lithium), the inhibitory effect of EIPA on the D-to-A transition was decreased. However, there is no direct evidence that amilorides inhibitors (i.e. EIPA) bind to antiporter-like subunits of complex I. The initial publication showing that amilorides protect ND5 subunits from covalent labeling by fenpyroximate [60] was later challenged by Miyoshi group [61]. Indeed, recently it has been demonstrated that amiloride derivatives bind in the quinone pocket of bovine complex near subunit 49 kDa [62].

The A/D transition of complex I may be considered a critical event in determining the outcome of ischemia/reperfusion [22,63,64]. In animal models, diminution of complex I activity during reoxygenation would protect mitochondria from irreversible damage [63,65–68] most likely via a decrease in oxidative stress [63,68,69]. Relatively slow activation of the D-form (during D-to-A transition) upon tissue reoxygenation may therefore serve as a protective valve and reduce the burst of respiration and consequently ROS production at the level of complex I or downstream sites. Therefore, the outcome of the reperfusion can be significantly influenced by the rate of activation of the D-form of the enzyme. As the cellular ionic balance changes dramatically during ischemia/reperfusion (see [70] for review), the significant rise in cytoplasmic sodium [70,71] changes sodium entry to mitochondria. Consequently, it would affect activation of complex I upon oxygen deprivation and reperfusion [72,73]. The effect of sodium on A/D transition, which is discussed here, might influence the rate of the D-to-A conversion of the mitochondrial complex I in pathological scenarios, providing another important mechanism for fine tuning of the activity of this enzyme.

## Transparency Document

Transparency document.

## Figures and Tables

**Table 1:** pH-dependence of the apparent activation rate constant of D-to-A transition (k_D → A_) in NADH:Q_1_ oxidoreductase reaction and the apparent dissociation constant of the D-form and cation.

pH		k_D → A_, min^− 1^^a^			K_D_, mM b		
control	Li^+^	Na^+^	Cs^+^	Li^+^	Na^+^	Cs^+^
7.0	1.24 ± 0.06	1.04 ± 0.10	1.76 ± 0.28	1.28 ± 0.12	723 ± 149	n.d.	601 ± 202
7.5	1.21 ± 0.22	0.85 ± 0.03	1.25 ± 0.09	0.83 ± 0.08	184 ± 62	n.d.	141 ± 41
8.0	1.13 ± 0.16	0.75 ± 0.12	0.95 ± 0.10	0.62 ± 0.07	201 ± 42	246 ± 65	168 ± 21
8.5	1.17 ± 0.18	0.51 ± 0.09	0.56 ± 0.06	0.36 ± 0.04	83 ± 5	137 ± 16	71 ± 5
9.0	1.15 ± 0.11	0.31 ± 0.05	0.31 ± 0.03	0.24 ± 0.05	85 ± 7	90 ± 9	45 ± 7

a Measured at 200 mM concentration of monovalent cation.

b Determined from the abscissa intercepts in Fig. 5
